# Catching Breath: The Making and Unmaking of Tuberculosis

**DOI:** 10.3201/eid2405.180185

**Published:** 2018-05

**Authors:** Margaret J. Oxtoby, Elizabeth M. Dufort

**Affiliations:** Centers for Disease Control and Prevention, Atlanta, Georgia, USA (M.J. Oxtoby);; New York State Department of Health, Albany, New York, USA (M.J. Oxtoby, E.M. Dufort)

**Keywords:** tuberculosis, mycobacterium infection, microbiology, history, bacteria, respiratory infections, mycobacterium

Tuberculosis (TB) is the leading infectious disease cause of death worldwide, yet many persons in industrialized countries think TB is mainly a historical curiosity or a rare disease only affecting a few uniquely vulnerable persons. This paradox is the inspiration for Kathryn Lougheed’s book, Catching Breath: The Making and Unmaking of Tuberculosis (Figure). After spending a decade at the bench exploring the mycobacterium as a microbiologist, the author embraced broader historical questions and embarked on a personal pilgrimage, as if learning about the disease for the first time. The result is, in her own words, a “whistle-stop tour through the past few million years to pick apart what has made TB ‘TB’.”

**Figure:**
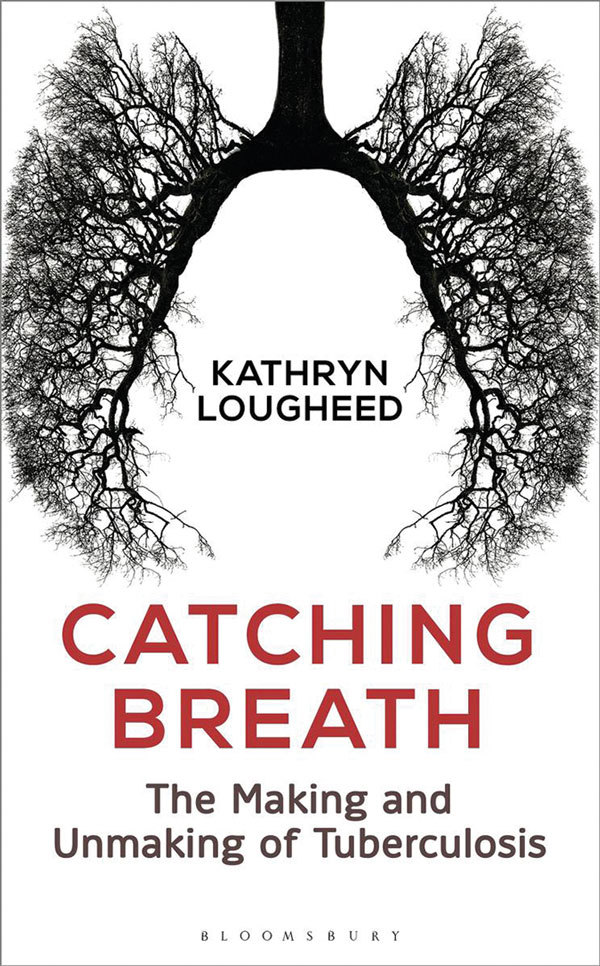
Catching Breath: The Making and Unmaking of Tuberculosis

Why is *Mycobacterium tuberculosis* still a dominant pathogen? Lougheed attempted to answer this question by exploring the ancient origins of mycobacterial infections and the expanding deadly toll of tuberculosis as human settlements grew many centuries ago. The scientific biography delves into the discovery of the microorganism at the end of the 19th century, the development of antimicrobial drugs active against the organism in the 20th century, and novel molecular techniques to detect TB genetic material and immunologic assays that have brought ever more sophisticated response to the disease. Yet Lougheed highlights that little dent has been made in TB’s enduring impact.

Whereas the book is most detailed in describing work closest to her own, the narrative extends beyond the laboratory to provoke new ways of thinking about the science of TB and assert the relevance of the humanistic and socioeconomic sides of TB. Lougheed does not shy away from the broader themes of economic inequality, political will, stigma, and interdisciplinary teamwork that affect each individual treatment and have driven and will continue to drive the epidemic’s long-term trajectory. Dr. Lougheed has marshaled an admirable sweep of complex data into a coherent and quickly moving narrative, peppering personal anecdotes throughout and bringing scenes to life. The language feels fresh, conversational, and opinionated, yet the explanations are grounded in sound research and are particularly detailed in areas related to microbiology. Her findings are often entertaining, startling, and moving.

The broad approach to such a complex topic might leave the reader wanting more clarity or depth in certain areas; however, the narrative is balanced by the enjoyment one gains from her storytelling with equal parts science, humor, history, microbiology, and real world contextualization. Readers, whether knowledgeable about tuberculosis or encountering it for the first time, will likely find that the story and the clearly posed questions deepen their interest and understanding and quicken their steps in pursuit of further answers.

